# Association between grip strength and shooting accuracy in male professional ice hockey players: an exploratory prospective pilot study

**DOI:** 10.1186/s13102-026-02235-1

**Published:** 2026-11-05

**Authors:** Daniel Muder, Mikael Swarén

**Affiliations:** 1 Department of Surgical Sciences, Orthopaedics and Hand Surgery, Uppsala University, Uppsala, Sweden; 2 Centre for Clinical Research Dalarna, Uppsala University, Falun, Sweden; 3 Swedish Unit for Metrology in Sports, Department of Sport, Fitness and Health, Dalarna University, Falun, Sweden

**Keywords:** Ice hockey, Grip strength, Shooting accuracy, Performance, Hand function, Elite athletes

## Abstract

**Objectives:**

To investigate whether grip strength is associated with shooting accuracy in professional ice hockey players and whether changes in grip strength are associated with changes in shooting accuracy following a supervised grip-strength training program.

**Methods:**

Nineteen male professional ice hockey players from a Swedish Hockey League team underwent baseline assessment of dominant-hand grip strength using a Jamar dynamometer and shooting accuracy using a standardized on-ice shooting test. Players then completed a 12-week supervised grip-strength training program integrated into their regular strength and conditioning schedule. Seventeen players completed follow-up testing. Associations were analyzed using Pearson correlations, linear regression, and paired-samples t-tests.

**Results:**

At baseline, grip strength showed a moderate positive correlation with shooting accuracy, which did not reach statistical significance (*r* = 0.452, *p* = 0.052; R² = 0.204). Following the intervention, grip strength increased from 54.4 ± 7.3 kg to 59.1 ± 6.5 kg (*p* < 0.001), while shooting accuracy improved from 23.9 ± 6.3% to 37.1 ± 7.2% (*p* < 0.001). Improvements in grip strength were positively associated with improvements in shooting accuracy (*r* = 0.599, *p* = 0.011; R²=0.359). In a linear regression analysis, changes in grip strength were positively associated with changes in shooting accuracy (regression coefficient = 1.335, *p* = 0.011).

**Conclusion:**

At baseline, grip strength showed a moderate, non-significant positive association with shooting accuracy in this exploratory prospective pilot study of professional ice hockey players. Improvements in grip strength were associated with improvements in shooting accuracy over the 12-week study period. These findings suggest that grip strength may represent a modifiable factor related to hockey-specific shooting performance. However, the absence of a control group and the continuation of regular team training preclude attributing the observed improvements specifically to the grip-strength programme. Larger controlled studies are warranted.

**Trial registration:**

The study was registered at ClinicalTrials.gov (NCT07115160), with the first submission meeting quality-control criteria on 4 August 2025 and the record first posted on 11 August 2025, prior to participant enrollment.

## Introduction

### Background

Ice hockey is a high-intensity intermittent collision sport requiring a complex interaction of technical skill, neuromuscular coordination, strength, power, and tactical decision-making [1]. In addition to lower-body performance, upper-extremity function plays an important role in puck control, stick handling, shooting execution, and physical battles along the boards [1]. Effective transfer of force through the upper extremity to the stick is essential for successful shot execution and may influence both shot velocity and shooting precision [2]. During shooting, the hands form the interface between the player and the stick. Grip force may contribute to maintaining a stable hand–stick connection while the upper extremities generate and transmit force and the player adjusts the stick’s position and orientation. Greater grip strength could therefore plausibly support stick control during shot execution. However, maximal grip strength is only one component of this complex task, which also depends on coordination, technique, and strength and power generated elsewhere in the body.

Grip strength is a simple, reliable, and widely used measure of upper-limb function and overall muscular strength. In both general and athletic populations, grip strength has been associated with physical capacity and performance-related characteristics [3–5]. Within ice hockey, grip strength has most commonly been used for physical profiling and monitoring purposes. Toong et al. [6] established normative grip-strength values in youth hockey players and demonstrated associations with age, body mass, sex, and playing level, suggesting that grip strength may reflect hockey-relevant physical capacity and may have utility as part of hockey-specific physical assessments. Grip strength was selected for the present study because it provides a practical and reproducible measure of the force-generating capacity of the hand, which directly interfaces with the hockey stick. Unlike broader upper-body strength assessments, handgrip dynamometry allows this specific physical characteristic to be assessed using a standardized procedure with minimal disruption to the players’ regular training schedule. This choice does not imply that grip strength is more important for shooting performance than other components of upper-body strength, power, or technical skill.

Previous research investigating shooting performance in ice hockey has largely focused on shot velocity, upper-body strength, power production, and training interventions. Bežák and Přidal [2] reported significant associations between upper-body strength, power, and shot velocity in professional and semi-professional hockey players, indicating that physical capacity contributes to hockey-specific shooting performance. Similarly, Novak et al. [7] demonstrated improvements in both shooting accuracy and handgrip strength following a shooting-training intervention in youth hockey players. Collectively, these findings suggest a potential relationship between upper-extremity function and shooting performance; however, the specific contribution of grip strength remains unclear. Studies in other stick- and bat-based sports suggest that grip strength may contribute to implement control and force transmission. Grip strength has been associated with shot velocity in lacrosse [8], whereas findings regarding bat swing velocity in baseball are mixed [9, 10]. However, these findings do not establish whether grip strength is associated with shooting accuracy in ice hockey.

Shooting accuracy represents a fundamental hockey skill directly related to offensive performance and scoring opportunities. Unlike broader performance metrics, shooting accuracy provides an objective and reproducible measure of technical execution that can be evaluated under standardized conditions. Given the biomechanical demands of puck control and stick stabilization during shooting, grip strength may plausibly contribute to shooting precision. However, evidence supporting this relationship in professional ice hockey players remains limited.

### Knowledge gap

Despite the theoretical importance of grip strength for stick control and shot execution, few studies have specifically examined the relationship between grip strength and shooting accuracy in ice hockey. Existing literature has primarily focused on physical profiling, normative grip-strength values, shot velocity, or general performance characteristics [2, 6]. Furthermore, although Novak et al. [7] reported concurrent improvements in shooting accuracy and handgrip strength following a training intervention in youth players, the relationship between changes in grip strength and changes in shooting performance was not directly evaluated.

Consequently, it remains unclear whether players with greater grip strength demonstrate superior shooting accuracy and whether improvements in grip strength are associated with improvements in shooting accuracy over time. As grip strength represents a potentially modifiable physical characteristic that can be measured rapidly and inexpensively, clarifying this relationship may provide insight into hockey-specific skill execution and inform future performance-oriented training strategies.

The present study therefore addresses two questions: whether grip strength is associated with shooting accuracy in professional ice hockey players and whether improvements in grip strength are associated with improvements in shooting accuracy over a 12-week training period. Addressing these questions may help clarify whether grip strength represents a potentially modifiable factor related to shooting performance in elite ice hockey.

### Aim

#### Primary aim

To explore the association between grip strength and shooting accuracy in male professional ice hockey players.

#### Secondary aim

To explore changes in grip strength and shooting accuracy over a 12-week period during which players undertook grip-specific training, and to examine the association between changes in grip strength and changes in shooting accuracy.

#### Hypotheses

We hypothesized that higher baseline grip strength would be associated with greater shooting accuracy and that both grip strength and shooting accuracy would improve during the intervention period. We further hypothesized that improvements in grip strength would be positively associated with improvements in shooting accuracy.

## Methods

### Study design

Exploratory prospective pilot study with baseline assessment and a subsequent supervised 12-week training period. The study was prospectively registered at ClinicalTrials.gov (NCT07115160).

### Patient and public involvement

Patients or members of the public were not involved in the design, conduct, reporting, or dissemination plans of this research.

### Participants

Participants were male professional ice hockey players aged 20–35 years at baseline, recruited from one Swedish Hockey League (SHL) team. Participants were recruited using convenience sampling from the available team roster. No participants were younger than 18 years at inclusion. The registered protocol initially envisaged 40 male and female players. However, practical recruitment constraints limited participation to one men’s SHL team. Eligible participants were professional ice hockey players signed with the participating SHL team who were free from current hand injury at the time of testing. Players with a current hand injury or any condition affecting their ability to perform maximal grip strength testing or the shooting assessment were excluded.

No formal a priori sample size calculation was performed; the final sample size was determined by the number of eligible players available for recruitment within the participating team. Nineteen players completed baseline testing. Seventeen players completed follow-up testing and were included in the longitudinal analyses. Two players were unavailable for follow-up assessment due to injury or personal circumstances and had transferred to other clubs before additional testing could be arranged.

The study was approved by the Swedish Ethical Review Authority (Etikprövningsmyndigheten), Dnr 2024-08560-01, and conducted in accordance with the Declaration of Helsinki. All participants provided written informed consent prior to study participation.

### Baseline testing

#### Grip strength

Grip strength was measured using a calibrated Jamar dynamometer according to the standardized testing procedures of the Swedish National Quality Registry for Hand Surgery (HAKIR) [11]. Participants were seated with the elbow close to the waist and flexed at approximately 90°, with the forearm and wrist in a neutral position. The dynamometer was set to the second handle position and supported by the examiner. Participants were instructed to squeeze the handle as hard as possible and to maintain the effort until the examiner instructed them to relax. Each effort lasted approximately 3 s, or until the dynamometer pointer no longer increased, indicating that the maximum reading had been reached. Testing alternated between the right and left hands, providing approximately 10–15 s of rest between successive measurements of the same hand. Three measurements were obtained for each hand, and the mean value of the dominant hand in kilograms was used for analysis. Hand dominance was self-reported by the participants. Dominant-hand grip strength was used in the association analyses, irrespective of whether that hand was positioned at the top or bottom of the hockey stick. Stick-hand position was recorded but was not included in the analyses. Grip strength was analyzed in absolute values (kg) and was not normalized to body mass.

#### Shooting performance

Shooting accuracy was assessed using a standardized on-ice protocol designed to quantify shooting precision under controlled conditions. Testing was conducted during a regular team practice session and before the shooting assessment, players completed the team’s regular pre-ice physical warm-up, followed by an on-ice warm-up involving skating, passing drills, and shooting. No additional study-specific warm-up was prescribed. Players then performed approximately 2–5 practice shots from the same position and towards the same target used for the 30 recorded shots. The same warm-up approach was used at both assessment sessions. All players wore their regular hockey equipment, used their own sticks, and performed wrist shots only. A standard SHL goal and a standard SHL puck were used, and no goalkeeper was present during testing.

Two circular targets (13 cm diameter) were positioned in the upper left and upper right corners of the goal. Players performed all shots from a fixed shooting position 6.0 m from the goal. The shooting distance was initially estimated at 4–5 m in the registered protocol. Before baseline data collection, a fixed distance of 6 m was selected as more practical and suitable for the on-ice testing environment. This distance was maintained at both baseline and follow-up assessments.

The test consisted of 30 wrist shots performed in alternating blocks of five shots toward the left and right targets until all attempts were completed. The starting side was determined by chance. A successful attempt was defined as direct puck contact with the target. Shooting accuracy was calculated as the percentage of successful hits out of 30 attempts.

**Fig. 1:**
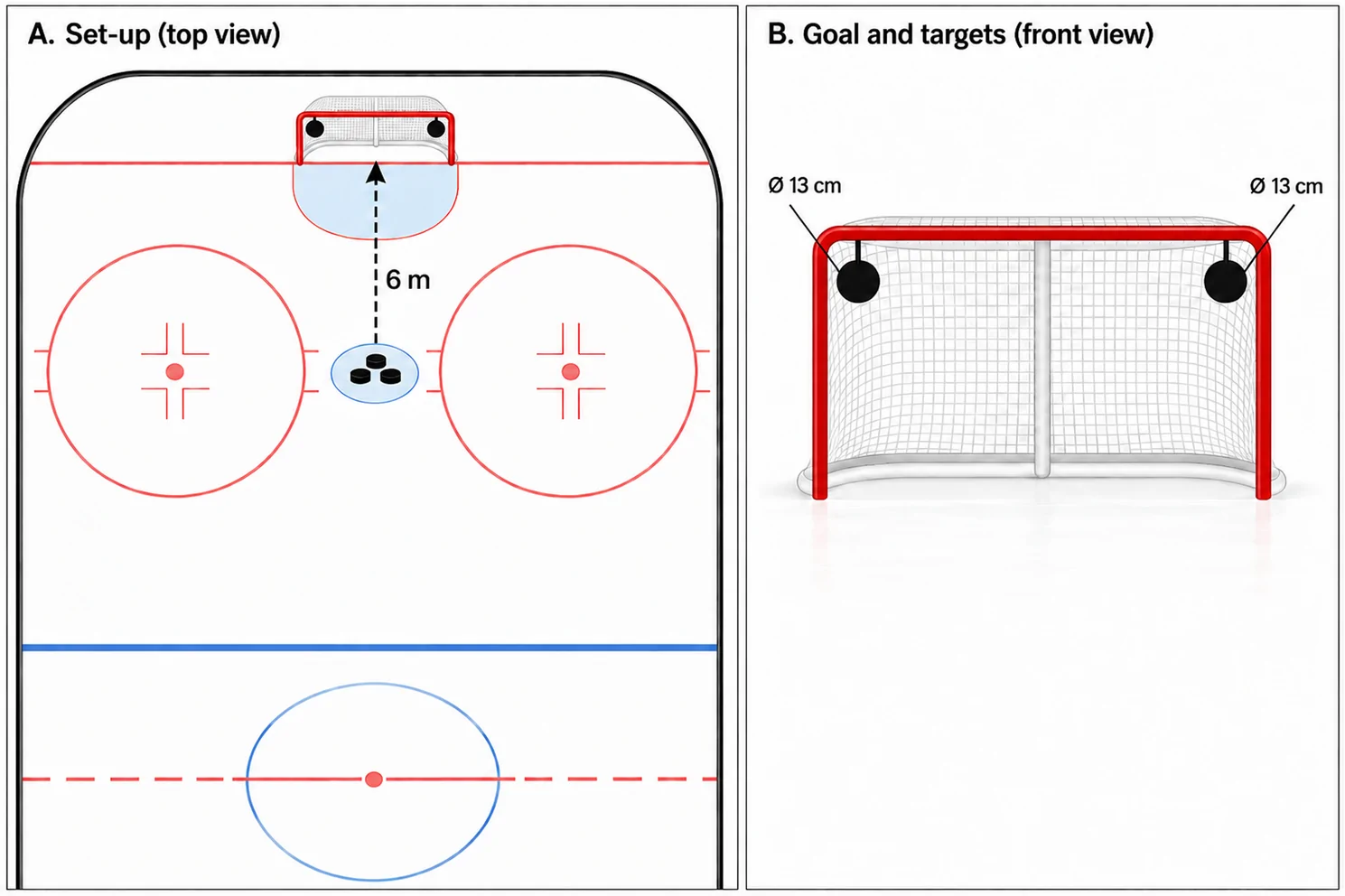
Schematic illustration of the shooting accuracy test. **A** Top view of the test setup. Players performed 30 wrist shots from a fixed position 6 m directly in front of the goal, centrally between the face-off dots. **B** Front view of the goal showing two circular targets (13 cm in diameter) suspended in the upper left and upper right corners. Shots were performed in six alternating blocks of five shots, with the starting side determined by chance, resulting in 15 shots at each target

The test setup is illustrated in Fig. 1.

Target hits were independently recorded by two observers and classified as either a hit or a miss. Inter-rater agreement was assessed in a sample of 60 shots using Cohen’s kappa statistic.

### Intervention

The intervention consisted of a 12-week progressive grip-strength training program conducted during the competitive season between the two assessment time points. Throughout this period, players continued their regular team training and competition schedule, typically comprising approximately 2–3 games per week. The usual training-day schedule included one on-ice session and one off-ice strength and conditioning session, with approximately one rest day per week. However, the number and distribution of training sessions varied according to match commitments, with fewer training sessions during weeks with three games. Individual overall training volume and external training load were not systematically recorded. Grip-strength exercises were incorporated into the team’s regular strength and conditioning program, with two sessions per week typically planned by the strength and conditioning coach. The frequency was reduced to one session in some weeks to accommodate the competitive match schedule. The exercises were initially instructed by the team’s strength and conditioning coach and subsequently performed by the players as part of their regular training routine. The coach was responsible for directing progression and regularly checked exercise execution, as with other exercises in the team’s training programme. Individual attendance at grip-specific sessions, completed training volume, and exercise loads were not systematically recorded. Consequently, the actual training exposure and adherence of individual players could not be quantified.

The grip-focused component comprised plate holds and wrist curls, selected to combine static grip loading with dynamic wrist training, with the aim of improving grip strength. Both exercises could be incorporated into the team’s regular strength and conditioning sessions using existing equipment. For plate holds, players were prescribed 2–3 sets of 15–30 s at approximately RPE 7–9. For wrist curls, players were prescribed 2–3 sets of 10–12 repetitions of both wrist flexion and extension at approximately RPE 7–8. The prescribed ranges were selected by the strength and conditioning coach as practical, individually adjustable training doses within the team’s regular programme, rather than according to a grip-training protocol specifically validated for professional ice hockey players.

Exercise resistance was individualized, with progression directed by the strength and conditioning coach according to each player’s performance and perceived exertion. The estimated duration of the grip-specific component was approximately 10–15 min per session, depending on rest intervals and the number of sets performed.

In addition to the grip-specific exercises, players continued their regular in-season strength training program, which included multi-joint barbell, dumbbell, and kettlebell exercises. Although not specifically designed to isolate grip strength, these compound movements likely contributed to overall forearm and hand strength development.

No predefined progression schedule was used, and individual changes in exercise load were not systematically recorded.

### Outcomes

Shooting accuracy, expressed as the percentage of successful target hits, was designated as the primary outcome in the present manuscript. Game Score was the primary outcome in the registered protocol. During study implementation, the focus shifted to shooting accuracy because its standardized assessment was performed directly by the research team, whereas the match-based Game Score measure was less straightforward to establish during data collection. This change in the primary outcome was not prespecified.

Secondary outcomes included grip strength (kg), the association between baseline grip strength and shooting accuracy, and the association between changes in grip strength and changes in shooting accuracy. Match-based outcomes were obtained for the same ten SHL games surrounding each assessment (five before and five after). Game Score reflected weighted offensive and defensive contributions at even strength, adjusted for ice time. Shot attempts included shots on goal, missed shots, and blocked shots and were expressed per game played. The number of games played was recorded for each participant and assessment period.

### Statistical analysis

Continuous variables are presented as mean ± standard deviation (SD), and categorical variables are presented as number and percentage. Normality of continuous variables was assessed using the Shapiro–Wilk test and visual inspection of distribution plots.

Baseline associations between dominant-hand grip strength and shooting accuracy were analyzed using Pearson correlation coefficients. The coefficient of determination (R²) was calculated to estimate the proportion of variance in shooting accuracy explained by grip strength.

Changes in grip strength and shooting accuracy from baseline to follow-up were analyzed using paired-samples t-tests. Mean differences are presented with 95% confidence intervals (CI). Associations between changes in grip strength and changes in shooting accuracy were analyzed using Pearson correlation coefficients.

Effect sizes for paired comparisons were calculated using Cohen’s d and interpreted using conventional thresholds of 0.2, 0.5, and 0.8 for small, medium, and large effects, respectively [12].

A linear regression analysis was performed to examine the association between changes in grip strength and changes in shooting accuracy. Changes in Game Score and shot attempts per game between the two assessment periods were analyzed using paired-samples t-tests. Associations between changes in grip strength and changes in these match-based outcomes were assessed using Pearson correlation coefficients. These analyses were exploratory.

Statistical significance was set at *p* < 0.05. All analyses were performed using IBM SPSS Statistics, version 30.0.0 (IBM Corp., Armonk, NY, USA).

## Results

### Baseline characteristics

A total of 19 male professional ice hockey players from one Swedish Hockey League team were included at baseline. Their mean age was 26.5 ± 4.4 years (range, 20–35 years). Baseline anthropometric characteristics, grip strength, shooting accuracy, playing position, shooting side, and hand dominance are presented in Table 1.

**Table 1:** Baseline characteristics of the study population (*n* = 19)

Characteristic	Value
Age, years	26.5 ± 4.4
Height, cm	183.0 ± 6.5
Weight, kg	85.8 ± 6.7
Grip strength, kg	54.0 ± 7.0
Shooting accuracy, %	23.0 ± 6.0
Position, n (%)	
Forward	12 (63)
Defense	7 (37)
Shooting side, n (%)	
Left	11 (58)
Right	8 (42)
Hand dominance, n (%)	
Right	15 (79)
Left	4 (21)

Data are presented as mean ± standard deviation or number (%)

Inter-rater agreement for shooting accuracy assessment was evaluated using Cohen’s kappa, yielding κ = 0.789 (*p* < 0.001) based on the evaluation of 60 shots.

### Association between baseline grip strength and shooting accuracy

**Fig. 2:**
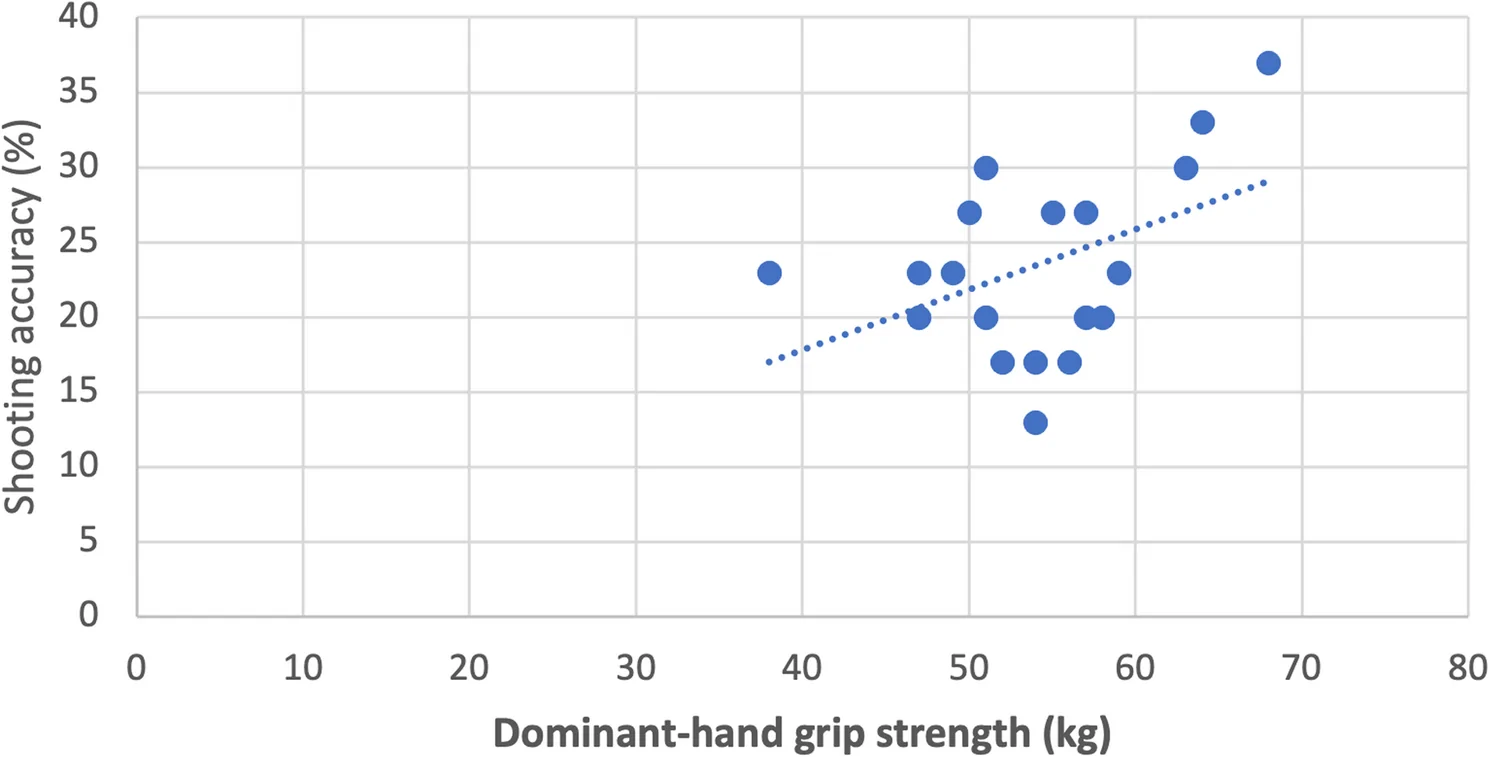
Association between baseline dominant-hand grip strength and shooting accuracy. Scatterplot showing the relationship between dominant-hand grip strength (kg) and shooting accuracy (%) at baseline in 19 male professional ice hockey players. Each point represents one player. The Pearson correlation coefficient was *r* = 0.452 (*p* = 0.052)

At baseline, dominant-hand grip strength demonstrated a moderate positive association with shooting accuracy (*r* = 0.452, *p* = 0.052; Fig. 2). Grip strength accounted for approximately 20% of the variance in shooting accuracy in the fitted model (R² = 0.204). The correlation was not statistically significant (*p* = 0.052), and the observed association should therefore be interpreted cautiously. In a sensitivity analysis restricted to the 17 players who completed both assessments, the baseline correlation was *r* = 0.518 (*p* = 0.033).

### Changes following the intervention

**Fig. 3:**
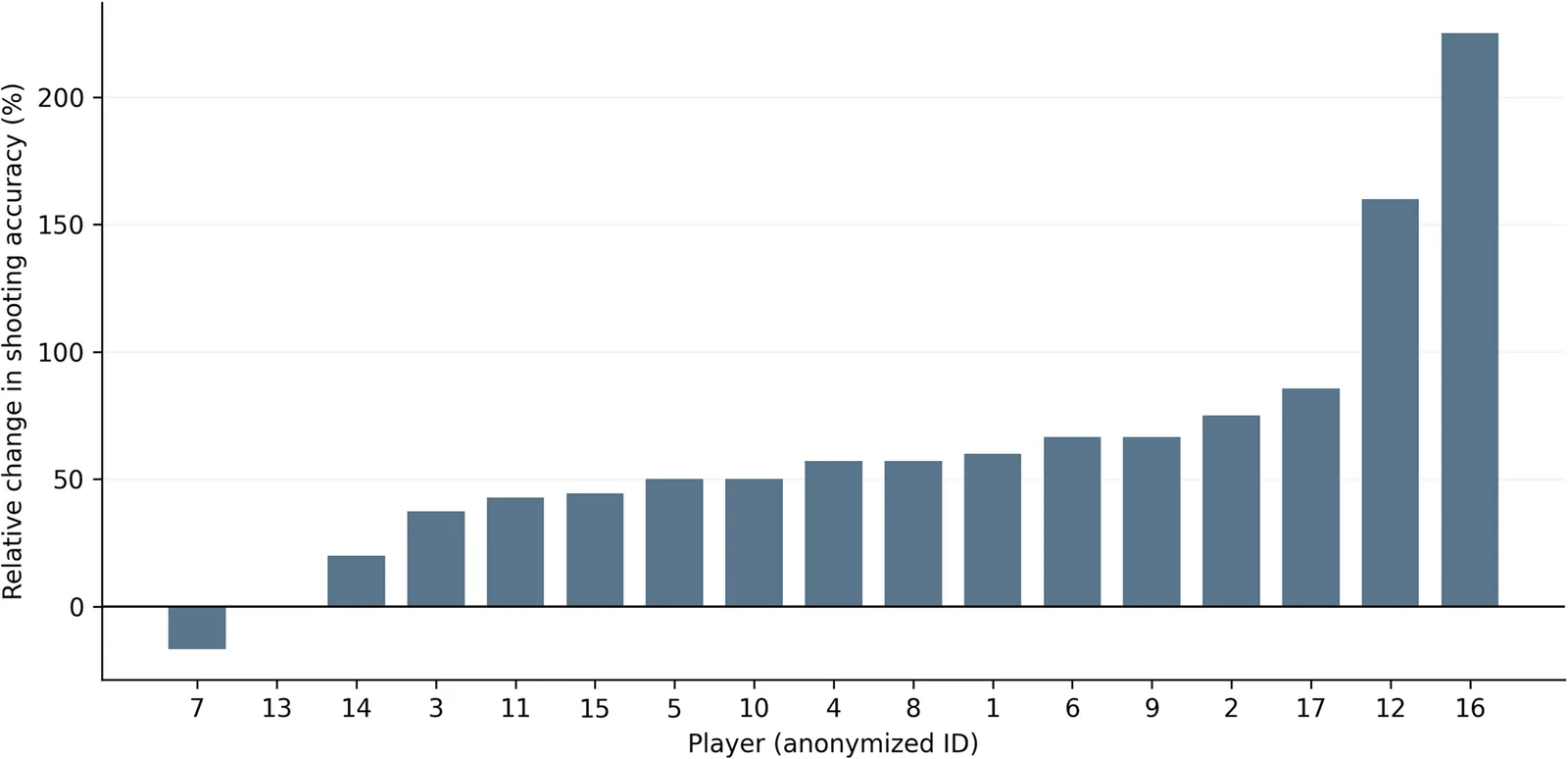
Individual relative changes in shooting accuracy from baseline to follow-up. Each bar represents one player (*n* = 17). Relative percentage change was calculated as [(number of target hits at follow-up − number of target hits at baseline) / number of target hits at baseline] × 100, based on 30 shots at each assessment. Players are ordered by magnitude of change

Seventeen players completed both baseline and follow-up assessments and were included in the longitudinal analyses. Two players were lost to follow-up due to injury or personal circumstances. Mean grip strength increased from 54.4 ± 7.3 kg at baseline to 59.1 ± 6.5 kg at follow-up, corresponding to a mean increase of 4.65 kg (95% CI, 2.73–6.56; *p* < 0.001; Cohen’s d = 1.25). Mean shooting accuracy increased from 23.9 ± 6.3% at baseline to 37.1 ± 7.2% at follow-up, corresponding to a mean increase of 13.12 percentage points (95% CI, 8.91–17.32; *p* < 0.001; Cohen’s d = 1.60) (Table 2; Fig. 3).

**Table 2:** Changes in grip strength and shooting accuracy following the 12-week intervention (*n* = 17)

Variable	Baseline	Follow-up	Mean change (95% CI)	*p*-value
Grip strength, kg	54.4 ± 7.3	59.1 ± 6.5	+ 4.65 (2.73 to 6.56)	< 0.001
Shooting accuracy, %	23.9 ± 6.3	37.1 ± 7.2	+ 13.12 (8.91 to 17.32)	< 0.001

Data are presented as mean ± SD unless otherwise specified

### Association between changes in grip strength and shooting accuracy

**Fig. 4:**
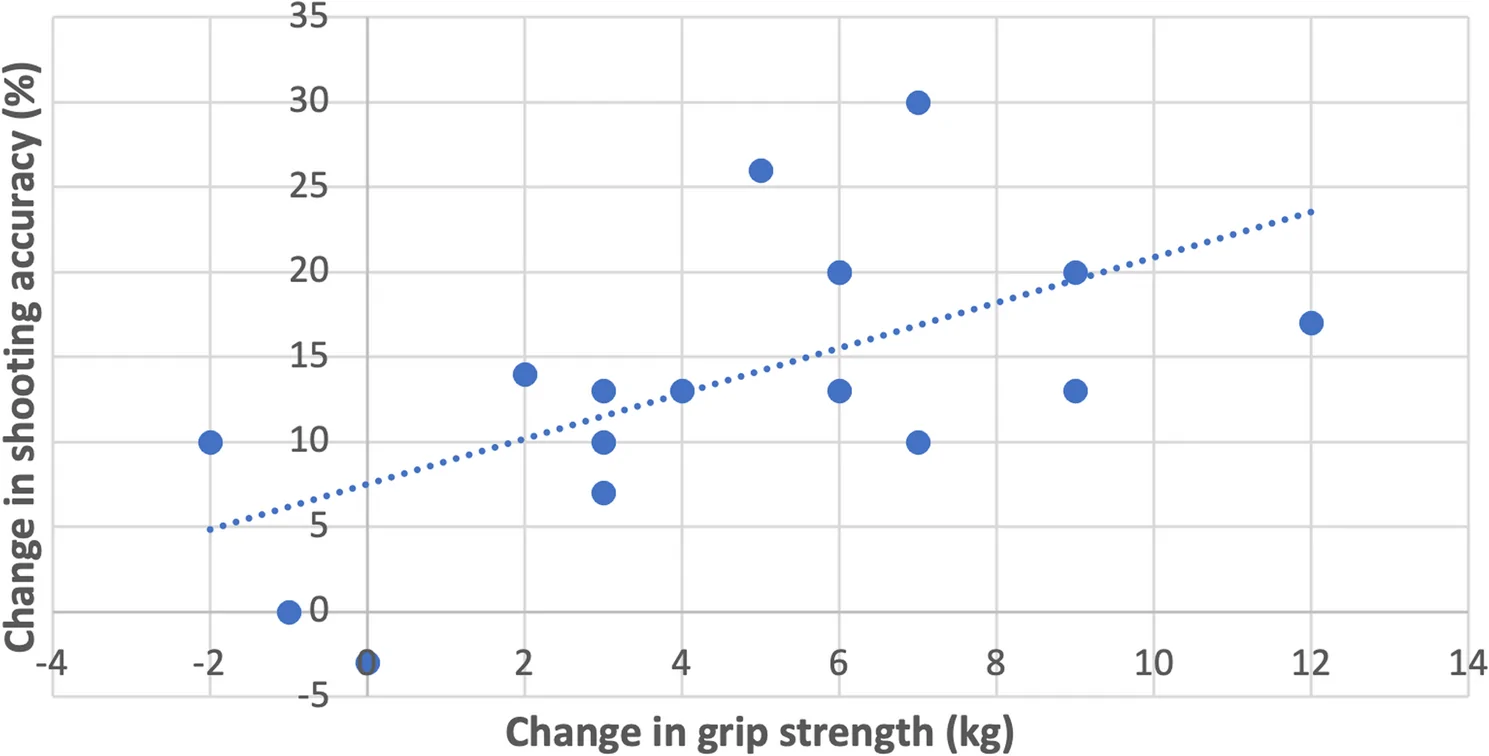
Association between changes in grip strength and changes in shooting accuracy. Scatterplot showing the relationship between changes in dominant-hand grip strength (kg) and changes in shooting accuracy (percentage points) from baseline to follow-up in 17 male professional ice hockey players. Each point represents one player. The Pearson correlation coefficient was *r* = 0.599 (*p* = 0.011)

Changes in grip strength were positively associated with changes in shooting accuracy following the intervention period (*r* = 0.599, *p* = 0.011; Fig. 4). Improvements in grip strength explained approximately 36% of the variance in improvements in shooting accuracy (R² = 0.359).

In a linear regression analysis, changes in grip strength were positively associated with changes in shooting accuracy (regression coefficient = 1.335, *p* = 0.011). The overall model explained 35.9% of the variance in shooting accuracy improvement.

Players demonstrating larger gains in grip strength generally exhibited greater improvements in shooting accuracy (Table 3).

**Table 3:** Associations between grip strength and shooting accuracy

Analysis	Coefficient	*R*²	*p*-value
Baseline grip strength vs. shooting accuracy	*r* = 0.452	0.204	0.052
Change in grip strength vs. change in shooting accuracy	*r* = 0.599	0.359	0.011
Linear regression: association between ΔGS and ΔAccuracy	Regression coefficient = 1.335	0.359	0.011

*ΔGS*  Change in grip strength, *ΔAccuracy*  Change in shooting accuracy

### Match-based outcomes

Among the 17 players who completed both assessments, Game Score was − 0.050 ± 0.190 at baseline and − 0.106 ± 0.206 at follow-up (mean ± SD; paired comparison, *p* = 0.222). Shot attempts per game were 2.03 ± 0.87 at baseline and 1.73 ± 0.69 at follow-up (*p* = 0.126). Changes in grip strength were not significantly correlated with changes in Game Score (*r* = 0.115, *p* = 0.660) or shot attempts per game (*r* = − 0.375, *p* = 0.138).

## Discussion

At baseline, grip strength demonstrated a moderate, albeit non-significant, positive association with shooting accuracy in professional ice hockey players. Although the baseline association did not reach conventional statistical significance, players with greater grip strength generally exhibited higher shooting accuracy. Furthermore, both grip strength and shooting accuracy improved during the 12-week intervention period. Most notably, improvements in grip strength were positively associated with improvements in shooting accuracy, suggesting that grip strength may represent a potentially modifiable factor related to shooting performance in elite ice hockey players. Given the small sample size and absence of a control group, these findings should be considered exploratory and hypothesis-generating.

Shooting performance in ice hockey is influenced by multiple interacting factors, including technical skill, neuromuscular coordination, lower- and upper-body strength, experience, and situational demands [13]. Consequently, grip strength alone is unlikely to account for a large proportion of shooting performance. Nevertheless, the moderate association observed at baseline and the stronger association between changes in grip strength and shooting accuracy suggest that grip strength may be relevant to shooting precision in elite players. The corresponding coefficients of determination were R² = 0.204 and R² = 0.359, respectively, accounting for approximately 20% and 36% of the observed variance in shooting accuracy at baseline and in changes in shooting accuracy. One potential explanation is that greater grip strength improves a player’s ability to stabilize and control the stick during shot execution. Grip-strength training may also induce neuromuscular adaptations, such as improved recruitment and coordination of the muscles involved in gripping and stabilizing the stick. These adaptations could potentially contribute to more consistent stick control during shooting. However, muscle activation, motor coordination, and shooting mechanics were not measured in the present study, and these mechanisms remain hypothetical. During shooting, the finger flexors contribute to gripping the stick, while the wrist flexors and extensors contribute to wrist positioning and stabilization [2]. A stronger grip may facilitate more consistent stick positioning and blade control at the moment of puck release, which could contribute to improved shooting accuracy. Although the present study did not assess shot mechanics directly, the observed relationship is biomechanically plausible and consistent with the known role of the upper extremity in hockey-specific skill execution.

Interestingly, the association between changes in grip strength and changes in shooting accuracy was stronger than the baseline association. This may suggest that within-player changes in grip strength are more relevant to shooting performance than between-player differences in absolute strength levels. Alternatively, the finding may reflect that grip strength serves as a marker of broader neuromuscular adaptations occurring during the intervention period. Given the exploratory nature of the study, these interpretations should be considered speculative and warrant further investigation.

Previous research in ice hockey provides context for the present findings but has generally examined outcomes other than the relationship between grip strength and shooting accuracy. Bežák and Přidal [2] reported associations between upper-body strength, power, and shot velocity in professional and semi-professional players. In contrast, our study assessed shooting accuracy rather than puck speed and found an association between changes in grip strength and changes in accuracy. Novak et al. [7] reported improvements in both handgrip strength and shooting accuracy following a shooting-training intervention in youth players. Although these findings concern similar outcomes, their intervention and study population differed from those of the present study, and they did not specifically examine the association between changes in the two measures.

Research in other implement-based sports offers a further point of comparison. Akiyama and Yamamoto [8] investigated the relationship between physical characteristics and shot velocity in lacrosse players, while Hughes et al. [9] examined grip strength, grip-strengthening exercises, and bat velocity in collegiate baseball players. Haruna et al. [10] examined strength and conditioning programs aimed at increasing bat swing velocity in collegiate baseball players. These studies address physical capacity and the performance of movements involving a stick or bat, but their velocity-based outcomes are not directly comparable with the shooting accuracy assessed in the present study. They therefore provide context for the possible relevance of physical characteristics to implement-based sporting skills rather than direct evidence that greater grip strength improves shooting precision.

In the present study, the baseline association between grip strength and shooting accuracy did not reach statistical significance, whereas changes in the two measures were positively associated over the 12-week period. This finding is exploratory and does not establish that grip-strength training caused the observed improvement in accuracy, particularly because players continued their regular team training and no control group was included. Shooting accuracy is likely to depend on several interacting physical and technical factors, consistent with previous research examining multiple off-ice physical characteristics in relation to on-ice hockey performance [13].

The present findings suggest that grip strength may represent a potentially modifiable and easily measurable physical characteristic related to shooting accuracy in professional ice hockey players. Because grip strength can be assessed rapidly using inexpensive and widely available equipment, it may serve as a useful component of athlete monitoring and performance assessment. Although the current findings are exploratory and do not establish a causal relationship, the observed associations warrant further investigation of grip-specific training as a potential strategy to support shooting performance. Given the multifactorial nature of hockey performance, grip strength should be considered one component of a broader physical and technical performance profile rather than a standalone determinant of shooting success.

Several limitations should be considered when interpreting the findings of this study. First, the sample size was relatively small and included players from a single professional team, which may limit the generalizability of the results. Consequently, the findings should be considered exploratory and hypothesis-generating. The small sample size also limits the precision of the estimated associations, and non-significant findings should not be interpreted as evidence that an association is absent.

Second, the study did not include a control group. Therefore, it cannot be determined whether the observed improvements in grip strength and shooting accuracy were attributable to the grip-strength intervention itself, normal training adaptations occurring throughout the season, or other unmeasured factors.

Third, a learning effect related to repeated exposure to the shooting test cannot be excluded. Although players completed familiarization shots before testing and the protocol was standardized across time points, improved familiarity with the testing procedure may have contributed to the observed improvements in shooting accuracy. Consequently, improvements observed during follow-up cannot be attributed solely to the grip-specific exercises and may partly reflect ongoing team training, seasonal adaptation, or familiarization with the shooting assessment. A ceiling effect is unlikely to explain the observed variability in shooting accuracy changes, as the highest baseline score was 11 of 30 successful shots (approximately 37%). However, the sensitivity of the shooting accuracy test to changes in performance has not been formally established.

Fourth, players continued their regular team training and strength-conditioning programs throughout the intervention period. While this reflects a real-world professional hockey environment, it limits the ability to isolate the specific effects of the grip-strength training component. In addition, individual adherence to the grip-strength program and the training dose actually completed by each player were not systematically recorded. Although the exercises were prescribed with target sets, repetitions or holding durations, and perceived effort, actual training volume, exercise tempo, and load progression could not be verified for individual players. This limits the reproducibility of the intervention and prevents assessment of whether differences in training exposure contributed to the observed outcomes.

Standardized shooting performance was assessed through shooting accuracy; puck velocity was not measured. Therefore potential trade-offs between shooting speed and shooting accuracy could not be evaluated. It remains possible that changes in shooting strategy or shot velocity contributed to the observed results. Future studies should incorporate simultaneous measurements of shooting accuracy and puck velocity to provide a more comprehensive assessment of shooting performance.

The study differed from the registered protocol in its smaller, male-only sample from a single team and in the designation of shooting accuracy rather than Game Score as the primary outcome. The change in outcome priority was not prespecified and should be considered when interpreting the findings. The available match-based shooting measure comprised all shot attempts rather than shots on goal alone, and the registered injury outcome was not collected. These deviations, together with the small sample size, limit the generalizability of the findings and warrant cautious interpretation.

Future studies should evaluate the relationship between grip strength and shooting performance in larger cohorts of elite ice hockey players and include appropriate control groups to better determine potential causal effects. Simultaneous assessment of shooting accuracy and puck velocity would provide a more comprehensive understanding of shooting performance and help clarify whether improvements in accuracy occur independently of changes in shot speed. Furthermore, biomechanical analyses of stick handling and shooting mechanics may help elucidate the mechanisms underlying the observed associations. Given the preliminary nature of the present findings, randomized controlled studies are warranted to determine whether targeted grip-strength interventions can meaningfully enhance hockey-specific performance outcomes.

## Conclusions

At baseline, grip strength demonstrated a moderate, non-significant positive association with shooting accuracy in professional ice hockey players. Improvements in grip strength were associated with improvements in shooting accuracy during a 12-week training period. These findings support further investigation of grip strength as a potentially modifiable factor related to hockey-specific performance. The absence of a control group and the continuation of regular team training prevent the observed improvements from being attributed specifically to the grip-strength programme.

## Data Availability

The datasets generated and analyzed during the current study are available from the corresponding author upon reasonable request, subject to applicable data protection and confidentiality regulations.

## Abbreviations

CI: Confidence Interval
HAKIR: Swedish National Quality Registry for Hand Surgery (Handkirurgiskt kvalitetsregister)
RPE: Rating of Perceived Exertion
SD: Standard Deviation
SHL: Swedish Hockey League
